# In situ spatial mapping resolves macrophage–fibroblast niche reorganisation in silica‐induced nodular lung fibrogenesis

**DOI:** 10.1002/ctm2.70869

**Published:** 2026-11-14

**Authors:** Lin Zhang, Jiaqi Tian, Ning Li, Qiang Li, Dandan Song, Xiaotan Shi, Shuyin Duan

**Affiliations:** ^1^ Clinical Research Center for Obstetrics and Gynecology Key Laboratory of Maternal & Fetal Medicine of National Health Commission of China Shandong Provincial Maternal and Child Health Care Hospital Affiliated to Qingdao University Jinan China; ^2^ Shandong Provincial Key Medical and Health Laboratory of Women's Occupational Exposure and Fertility Preservation Jinan China; ^3^ School of Public Health Shandong First Medical University Jinan China

Dear Editor,

Silicosis is an intractable occupational pneumoconiosis characterised by discrete, sharply demarcated fibrotic nodules that are pathologically distinct from the diffuse parenchymal remodelling of idiopathic pulmonary fibrosis.^1^, ^2^ Although spot‐based spatial transcriptomics has mapped cellular compositions in silica‐exposed lungs via computational deconvolution, resolving intra‐lesional cellular architecture across progressive stages requires subcellular in situ profiling.^3^, ^4^ Here, we deployed high‐plex in situ spatial transcriptomics (10× Genomics Xenium, 5006 gene targets)^5^ to profile 423 673 single cells on a single tissue microarray comprising lung sections from unexposed controls (day 0) and silica‐exposed mice at days 28 and 56 (*n* = 2 biological replicates per time point, six sections in total; Figure S1). Across this longitudinal series, individual single cells, segmented lesions and grid tiles served as multi‐scale spatial sampling units to quantitatively track the microenvironmental evolution of nodular fibrogenesis.

To objectively define nodule boundaries directly from transcriptional data, we developed the Multi‐Evidence Nodule Score (MENS) coupling local fibrosis‐associated cell density with marker gene expression (*Cd68*, *Col1a1*, *Fn1* and *Tgfb1*; Figure 1a,d,e).^6^ Applying a section‐specific two‐component Gaussian mixture cutoff and requiring an inflammatory infiltrate in candidate regions ensured that segmented lesion burden reflected bona fide disease progression, demonstrating that the share of cells within nodules expanded from 8.9% and 11.2% at day 28 to 19.8% and 23.4% at day 56 (Figure 1b). In control sections, the cutoff alone marked 21.2% and 17.6% of cells along smooth muscle‐rich bronchovascular bundles, but these candidates held 0.06% and 0.11% *Trem2* ^+^/*Gpnmb* ^+^/*Mmp12* ^+^ macrophages (hereafter, *Trem2* ^+^ macrophages) and fewer lymphocytes than adjacent parenchyma, so no lesion was called (Figure 1b). Since nodular cells carried more transcripts than parenchymal cells of the same section (Figure 1c), we therefore tested the boundary against a transcript‐free measure. Morphology‐derived nuclear density, computed from segmentation masks without transcript input, separated lesion tiles from parenchyma with areas under the receiver operating characteristic (ROC) curve (AUC) of 0.756 at day 28 and 0.819 at day 56 (Figure S2). Iterative leave‐one‐feature‐out sensitivity analyses verified that nodular spatial delineation was robust against circular feature selection: under an entirely macrophage‐free formulation omitting *Cd68* and all macrophage subsets, *Trem2* ^+^ macrophages remained enriched within nodules by 2.40 log_2_ units over parenchyma, accounting for 21.7% of intra‐nodular cells (compared to 3.11 log_2_ units and 28.5% under the unfiltered complete model; Figures 1f–h and S3).

**FIGURE 1:**
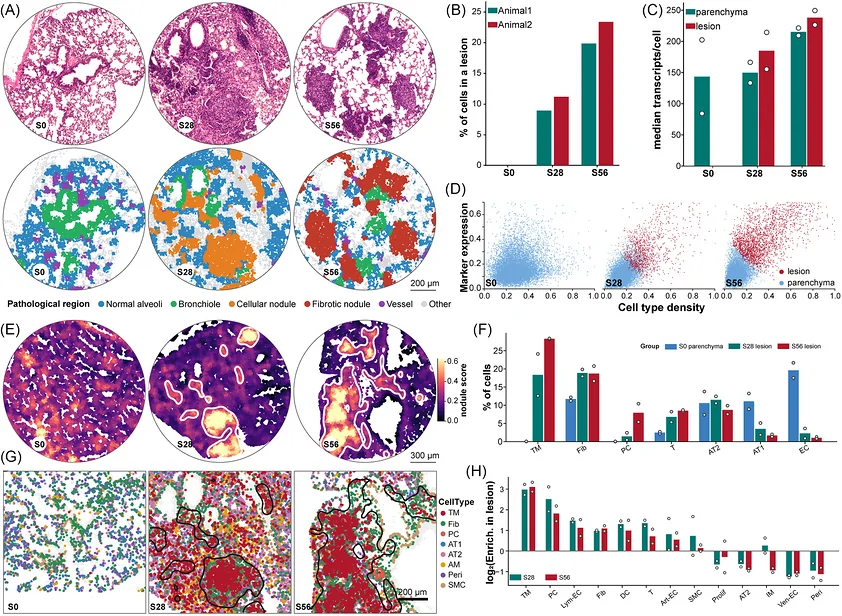
Silica exposure induces progressive expansion of transcriptionally defined fibrotic lesions enriched with *Trem2* ^+^ macrophages (TMs). Male C57BL/6 mice received a single intratracheal dose of 5 mg silica particles and were sacrificed at day 28 or 56, with saline‐treated mice serving as day 0 controls (*n* = 2 biological replicates per time point, six sections in total). (a) Representative haematoxylin and eosin (H&E) staining and corresponding computed pathological region maps across exposure stages. (b) Quantitative proportion of total cells located within segmented lesions across individual tissue sections. (c) Median transcript counts per cell compared between uninvolved parenchyma and nodular lesions. (d) Single‐cell marker gene expression (*Cd68*, *Col1a1*, *Fn1* and *Tgfb1*) plotted against local cell density in randomly sampled cells, stratified by lesion status. (e) Spatial Multi‐Evidence Nodule Score (MENS) heatmaps with segmented lesion boundaries outlined in white. (f) Cellular lineage composition compared between control parenchyma and silica‐induced lesions at days 28 and 56. (g) High‐magnification spatial maps centred on the primary lesion core coloured by cell type with lesion perimeters outlined in black. (h) Log_2_ fold enrichment of individual cell types within lesions relative to surrounding parenchyma. Bars represent the mean of *n* = 2 biological replicates per stage, with individual circles indicating section‐level values (c, f and h). Day 0 control sections harbour no lesions (b), with corresponding panels showing parenchymal tissue baseline (c–e, g and h). Images represent individual sections (a, e and g). Scale bars: 200 µm (a and g) and 300 µm (e). *n* = 2 mice per stage. AM, alveolar macrophages; AT1 and AT2, alveolar type 1 and type 2 epithelial cells; Art‐EC, Lym‐EC and Ven‐EC, arterial, lymphatic and venous endothelial cells; DC, dendritic cells; EC, endothelial cells; Fib, fibroblasts; IM, interstitial macrophages; PC, plasma cells; Peri, pericytes; Prolif, proliferating cells; SMC, smooth muscle cells; T, T cells; S0, S28 and S56, day 0, day 28 and day 56 post‐exposure.

Spatial niche dissection demonstrated cellular reorganisation across progressive disease stages (Figure S4). *Trem2* ^+^ macrophages expanded from 0.44% and 1.11% of total macrophages in control sections to 46.9% and 47.1% at day 56, emerging as the dominant intra‐nodular population (28.5% and 28.7% of nodule cells; Figure 2a–d). Consistently, this transcriptional phenotype mirrors the *Spp1* ^+^ profibrotic macrophage state recently identified inside silicotic lesions^4^ and conserved across organ fibrosis.^7^, ^8^ In mature day 56 lesions, *Trem2* ^+^ macrophages exhibited the highest enrichment over uninvolved parenchyma (3.10 log_2_ units; Figure S5), followed by plasma cells (1.81 log_2_ units), with fibroblasts also enriched (1.09 log_2_ units; Figure 1f,h). High‐resolution radial mapping identified a distinct concentric architecture within individual nodules, with *Trem2* ^+^ macrophages concentrating within the dense fibrotic core (mean normalised radius of 0.51 from the centroid at both exposed stages), whereas homeostatic alveolar macrophages were progressively restricted to the nodular periphery and adjacent alveolar spaces (Figures 2e–h and S6). In contrast to the diffuse airspace dispersal of macrophages in human fibrotic lungs,^9^ silica‐exposed macrophages organise into an in situ core‐to‐periphery gradient analogous to pulmonary granulomatous niches of patients with chronic sarcoidosis.^10^

**FIGURE 2:**
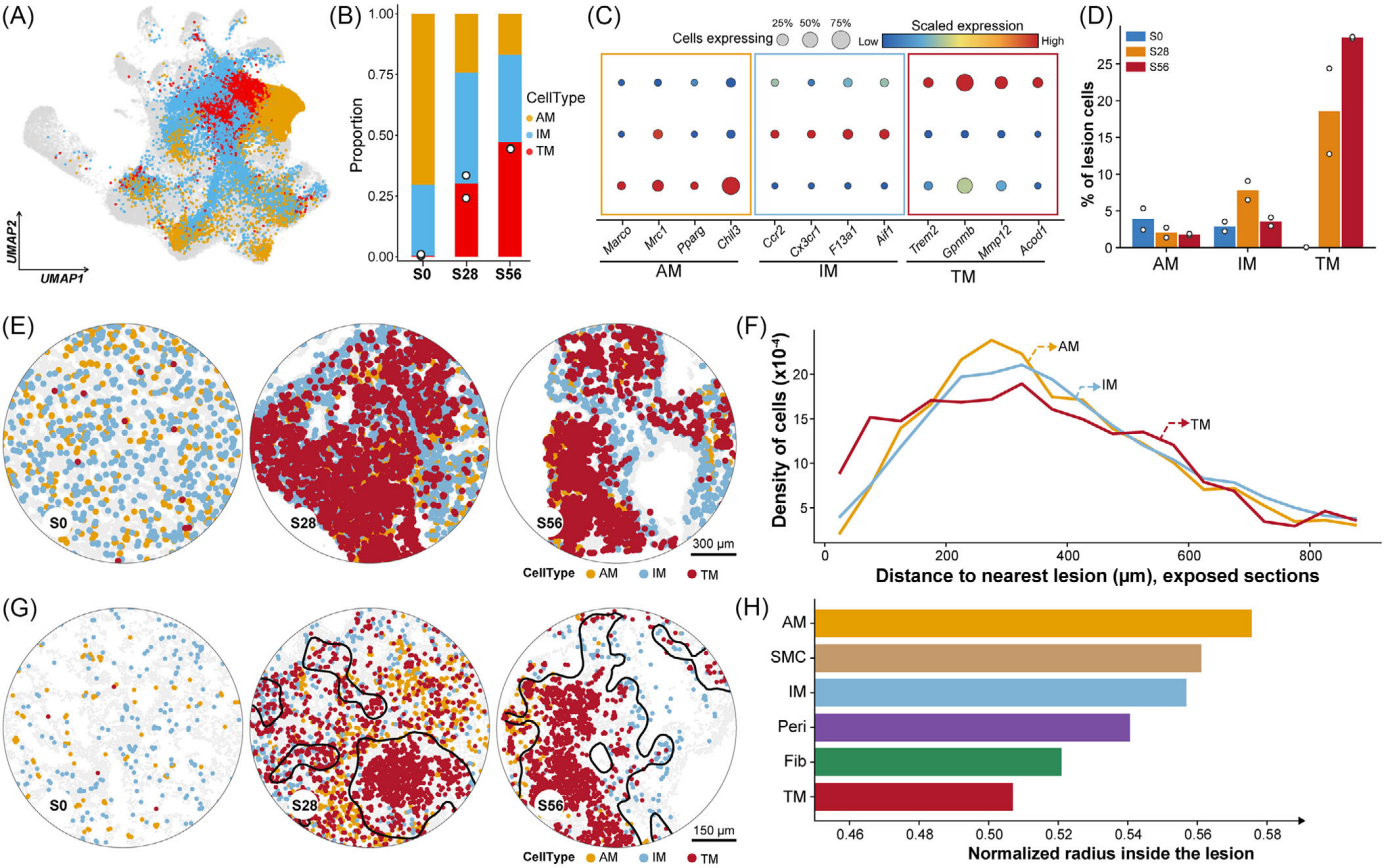
*Trem2* ^+^ macrophages (TMs) expand during silicosis progression and concentrate within the nodular core. (a) Uniform manifold approximation and projection (UMAP) plot of all profiled macrophages, highlighting three distinct macrophage sub‐lineages. (b) Relative macrophage sub‐lineage proportions across exposure stages (mean of *n* = 2 biological replicates per stage). (c) Marker gene expression profiles across macrophage sub‐lineages, with lineage‐defining markers boxed. Dot diameter indicates the percentage of expressing cells, and colour intensity reflects mean expression scaled across sub‐lineages. (d) Proportion of macrophage sub‐lineages within nodular lesions relative to total cells in control tissue. (e) Spatial distribution maps of macrophage sub‐lineages in representative lung sections across stages. (f) Radial distance of individual macrophages to the nearest lesion centroid, pooled across the four exposed sections (days 28 and 56). (g) Representative spatial fields centred on the primary lesion core with nodular boundaries outlined in black. (h) Mean normalised radial distribution of six major cell types inside lesions at days 28 and 56, scaled from lesion centroid (0) to 95th‐percentile radius (1.0). Bars represent the mean of *n* = 2 biological replicates, and circles denote individual section data (d). Axis in (h) is truncated at.45. Scale bars: 300 µm (e) and 150 µm (g). *n* = 2 mice per stage. AM, alveolar macrophages; Fib, fibroblasts; IM, interstitial macrophages; Peri, pericytes; SMC, smooth muscle cells; S0, S28 and S56, day 0, day 28 and day 56.

Composite pathway scoring localised transcriptional signatures for extracellular matrix remodelling, transforming growth factor‐beta (TGF‐β) and NOD‐, LRR‐ and pyrin domain‐containing protein 3 (NLRP3) inflammasome signalling specifically within nodular regions across both exposure stages (Figure 3a–c). Because *Trem2* ^+^ macrophages and fibroblasts co‐accumulate within these active lesions (Figure 3d), whole‐tissue coordinate shuffling yielded an apparent spatial proximity between the two populations, with the fibroblast and pericyte fraction among the 10 nearest neighbours of *Trem2* ^+^ macrophages reaching 1.14 times expectation at day 56 (Figure 3e,f). However, when tested against a compartment‐constrained null model that permuted cell labels strictly within nodule and parenchymal boundaries independently, the observed‐to‐expected ratio declined to 0.93 (empirical *p* = 1.000; Figures 3f,g and S7). Restricting permutations to intra‐nodular cells yielded neighbour ratios of 0.95 at day 28 and 0.89 at day 56, and the compartment‐constrained ratio remained at or below 1.00 across interaction radii (25–100 µm) and neighbour numbers (*k* = 5–30). Conditioned on lesion boundaries, cell‐resolved coordinate testing reveals that shared tissue compartmentalisation drives macrophage–mesenchymal co‐localisation within random spatial expectations.

**FIGURE 3:**
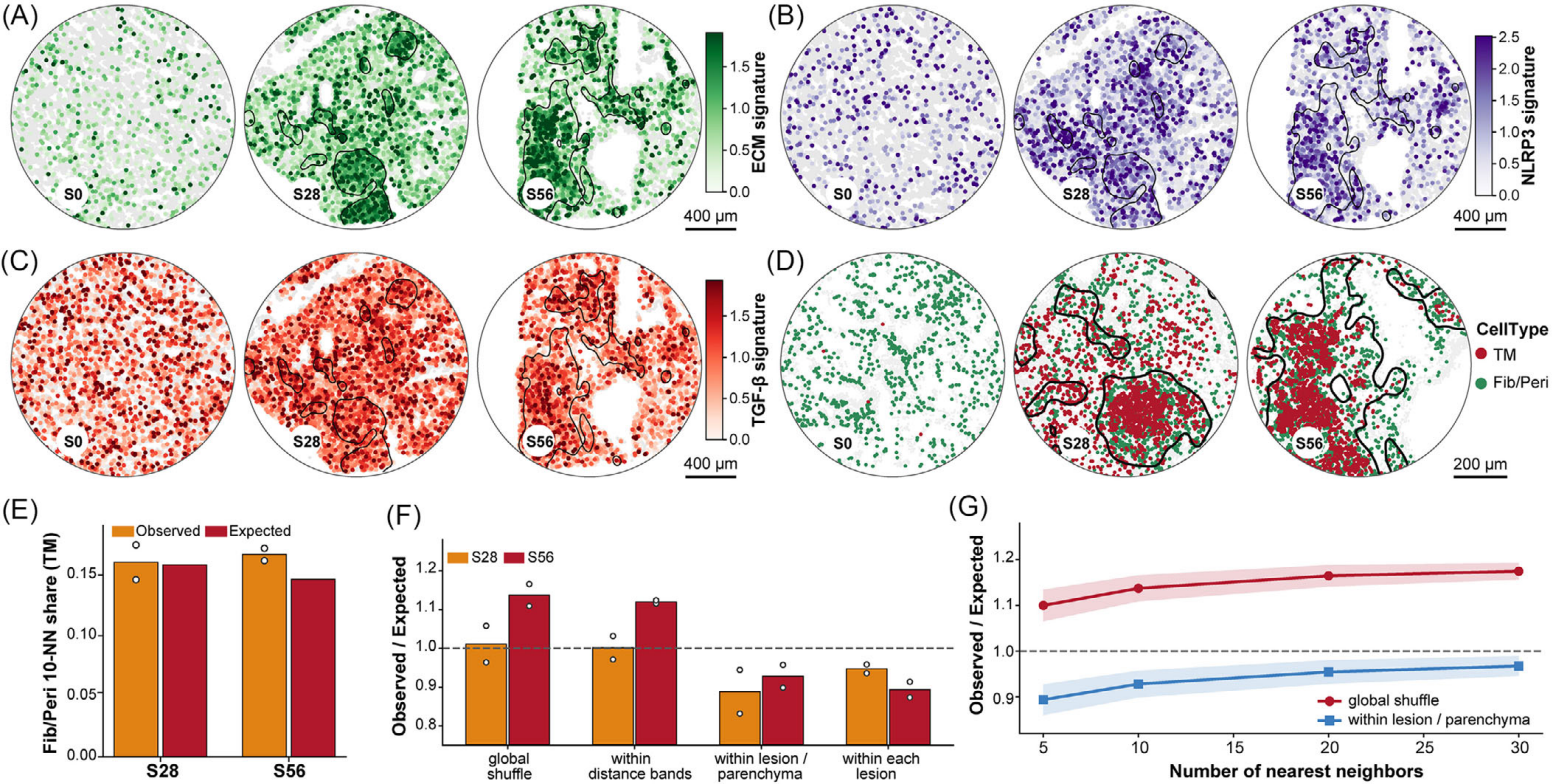
Compartment‐constrained spatial null models confirm random *Trem2* ^+^ macrophage (TM)–fibroblast proximity within lesions. (a–c) Single‐cell activity scores for extracellular matrix remodelling (a), NLRP3 inflammasome (b) and TGF‐β (c) signalling pathways with lesion perimeters outlined in black. (d) Representative spatial fields centred on the primary lesion core illustrating the co‐localisation of TMs with fibroblasts and pericytes. (e) Fibroblast and pericyte representation among the 10 nearest neighbours of TMs comparing observed spatial values against a global unconstrained permutation model. (f) Observed‐to‐expected ratio of fibroblast and pericyte representation evaluated across four distinct spatial null models. (g) Observed‐to‐expected neighbour ratios across varying neighbour numbers (*k* = 5‒30) in day 56 sections under global versus compartment‐constrained nulls. Constrained permutation models independently permute cell labels strictly within lesion and parenchymal compartments, individual lesions or concentric distance bands surrounding centroids (f and g). Dashed horizontal lines denote theoretical random expectation (f and g). Trend lines represent stage means with shaded bands spanning replicate values (g); unexposed control sections contain fewer than 50 TMs and are excluded from spatial neighbour analyses (e–g). Scale bars: 400 µm (a–c) and 200 µm (d). Observed statistics are compared with 500 (e and f) or 200 (g) label permutations per null model, and one‐sided empirical *p* equals the number of permutations at or above the observed value plus one, divided by the permutation count plus one. *n* = 2 mice per stage. ECM, extracellular matrix; Fib/Peri, fibroblasts and pericytes; S0, S28 and S56, day 0, day 28 and day 56.

Microenvironmental remodelling extended beyond the discrete nodular boundary into surrounding parenchyma. We formulated an exploratory alveolar health index (AHI) combining AT1 coverage, log‐transformed AT2/AT1 ratios, and immune infiltration across non‐overlapping 200 µm grid tiles (Figure 4a–c). Mean AHI declined from 0.515 and 0.606 in unexposed lungs to 0.411 and 0.322 at day 56, retaining consistent stage‐wise ordering across six weighting schemes (Figures 4d,h and S8). Perinodular tiles exhibited continuous radial remodelling gradients, with alveolar integrity recovering as a function of physical distance from the nearest lesion centroid at both exposed stages (Figure 4e–g). Methodologically, the spatial distance quantification provides a radial complement to temporal pseudotime trajectories constructed in diffuse fibrosis,^9^ capturing lesion‐centred disease expansion unique to nodular pneumoconiosis.

**FIGURE 4:**
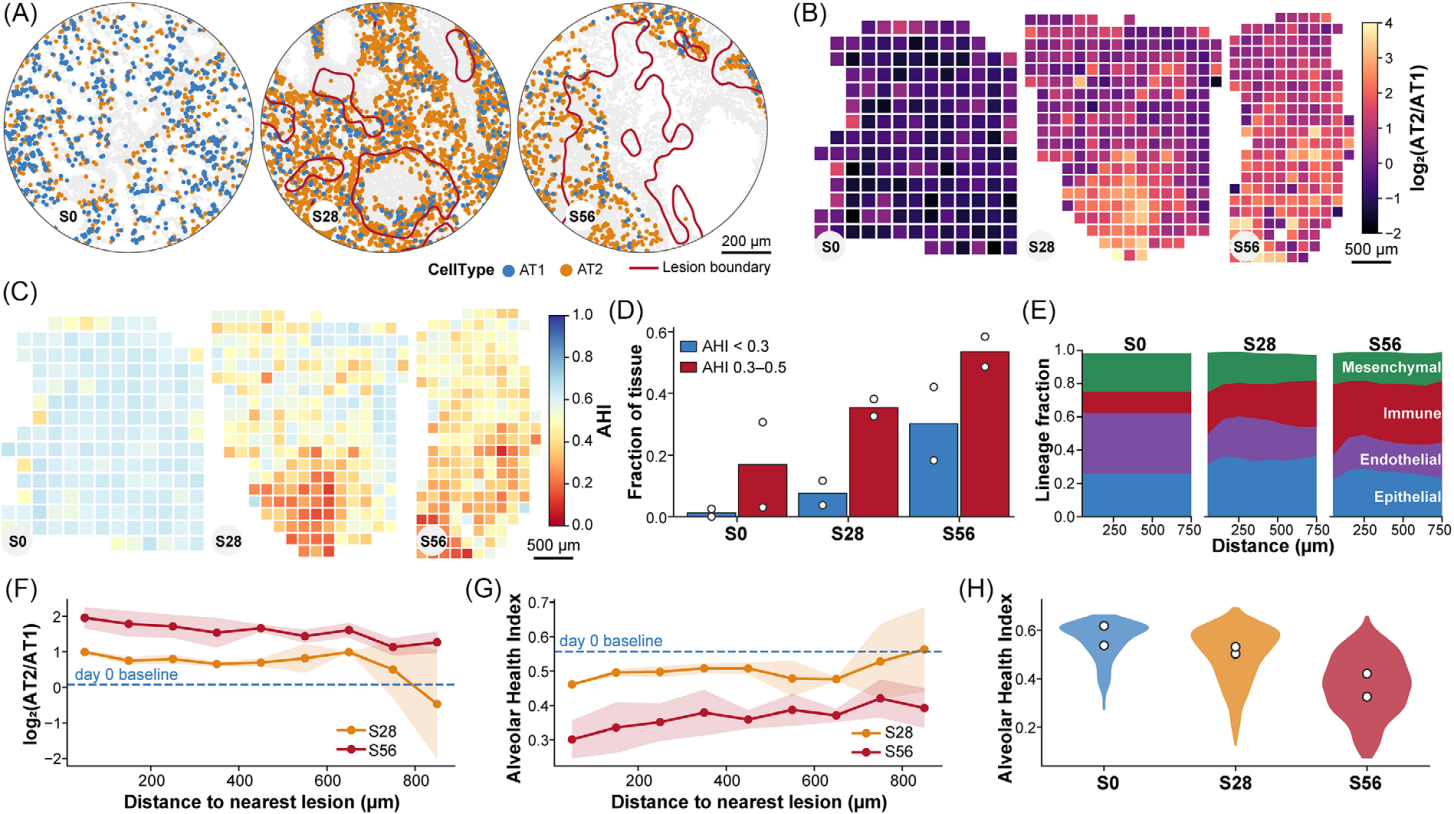
Silica exposure compromises alveolar health along a distance‐dependent radial gradient centred on fibrotic lesions. (a) Spatial organisation of alveolar type 1 and type 2 (AT1 and AT2) epithelial cells within fields centred on primary lesion cores with red outlines denoting lesion boundaries. (b and c) Quantitative spatial maps of log_2_‐transformed AT2/AT1 cell ratio (b) and alveolar health index (AHI) (c) computed across non‐overlapping 200 µm grid tiles. (d) Proportion of grid tiles categorised within the two lowest AHI severity tiers across disease stages. (e) Cellular lineage composition plotted as a continuous function of radial distance from the nearest lesion centroid with day 0 control baseline displayed for comparison. (f and g) Radial spatial profiles showing log_2_(AT2/AT1) ratios (f) and AHI values (g) of grid tiles relative to distance from the nearest lesion centroid. (h) Distribution of AHI values across all tiles per stage, with circles marking section medians. Bars represent the mean of *n* = 2 biological replicates, and circles denote individual section values (d). Lines in (f and g) display mean values across *n* = 2 sections with shaded regions spanning replicate bounds; dashed horizontal lines indicate day 0 control baseline. Unexposed control sections harbour no lesions and are omitted from radial distance analyses (f and g; analysing 576 tiles at days 28 and 316 tiles at day 56 within 900 µm of lesion centroids). Scale bars: 200 µm (a) and 500 µm (b and c). *n* = 2 mice per stage. S0, S28 and S56, day 0, day 28 and day 56.

In conclusion, *Trem2* ^+^ macrophages expand and concentrate within the core of silicotic nodules across the silica time course, while their proximity to fibroblasts inside lesions stays at random expectation under compartment‐constrained null models. Nodular compartmentalisation thus emerges as the primary organising feature of the silicotic lesion, and compartment‐aware null models provide the appropriate test for intercellular organisation in focal lung fibrosis.

## AUTHOR CONTRIBUTIONS

*Writing—original draft, resources, funding acquisition and conceptualisation*: Lin Zhang. *Writing—review and editing, supervision, project administration and conceptualisation*: Shuyin Duan. *Writing—review and editing, investigation, funding acquisition, methodology, validation and data curation*: Ning Li. *Writing—review and editing and investigation*: Jiaqi Tian, Qiang Li, Dandan Song and Xiaotan Shi.

## CONFLICT OF INTEREST STATEMENT

The authors declare they have no conflicts of interest.

## ETHICS STATEMENT

All animal experiments were approved by the institutional animal ethics committee of Shandong Provincial Maternal and Child Health Care Hospital (no. 2022‐1) and were performed in accordance with national guidelines for the care and use of laboratory animals. Consent for publication is not applicable, and no clinical trial is reported.

## Supporting information



Supporting Information

## Data Availability

Analysis code for the revision, including the leave‐one‐feature‐out nodule definition, the spatially constrained permutation tests and the morphology‐only validation, is version‐controlled and archived on Zenodo (https://doi.org/10.5281/zenodo.22934685).
