# Supplementary material for: In situ spatial mapping resolves macrophage–fibroblast niche reorganisation in silica‐induced nodular lung fibrogenesis
Source: Clin Transl Med. 2026 Nov 14;16(11):e70869. doi: 10.1002/ctm2.70869 (PMC13770215; doi:10.1002/ctm2.70869)
Supplement: Supplementary file 1 [file CTM2-16-e70869-s001.docx]

**Supplementary Figures**

**Supplementary Fig. S1. Subcellular Xenium *in situ* spatial profiling resolves 22 distinct cell lineages across murine silicosis progression.** **(a)** Schematic representation of experimental design and spatial transcriptomic analysis workflow. **(b)** UMAP visualization of 423,673 profiled single cells colored by annotated cell type (left) and major lineage (right). **(c)** Proportional cellular composition across individual tissue sections. **(d)** Whole-section spatial distribution maps of annotated cell lineages in representative lungs across exposure stages. **(e)** Total single-cell counts enumerated per lineage across all analyzed sections. **(f)** *In situ* spatial expression patterns of canonical cell-type marker genes in a representative day 56 lung section. Scale bars, 300 µm (d) and 1,000 µm (f). n = 2 biological replicates per stage. AM, alveolar macrophages; AT1 and AT2, alveolar type 1 and type 2 cells; B, B cells; Cil, ciliated cells; Art-EC, Cap-EC, Lym-EC, and Ven-EC, arterial, capillary, lymphatic, and venous endothelial cells; CM, cardiomyocytes; DC, dendritic cells; EC, endothelial cells; EC-lowQC, low-quality endothelial cells; Fib, fibroblasts; IM, interstitial macrophages; Meso, mesothelial cells; NA, unassigned; PC, plasma cells; Peri, pericytes; Prolif, proliferating cells; SMC, smooth muscle cells; T, T cells; TM, *Trem2*^+^ macrophages; S0, S28, and S56, day 0, day 28, and day 56.

**Supplementary Fig. S2. Morphology-derived nuclear density distinguishes fibrotic lesions from surrounding parenchymal tissue.** **(a)** Receiver operating characteristic (ROC) curves evaluating nuclear density as a binary classifier for lesion tiles across individual exposed sections. **(b, c)** Area under the ROC curve (AUC) values of three morphology-derived mask features for full-score lesions (b) and candidate lesions evaluated under a macrophage-free model (c). **(d)** Quantitative nuclear density compared between parenchymal and lesion grid tiles (100 µm tile size) across stages, with bars indicating median values. Bars represent the mean of n = 2 biological replicates with circles marking individual section data (b, c); dashed horizontal lines represent random classification baseline (AUC = 0.50; a–c). n = 2 mice per stage. S0, S28, and S56, day 0, day 28, and day 56.

**Supplementary Fig. S3. Intra-nodular *Trem2*^+^ macrophage enrichment remains robust under a macrophage-free lesion segmentation model.** **(a)** Pearson correlation of the density component, transcriptional component, and combined MENS score relative to the baseline full model across sections. **(b)** Jaccard similarity index comparing lesion segmentations generated by score variants against the full model in silica-exposed lungs. **(c)** Log_2_ fold enrichment of *Trem2*^+^ macrophages inside nodules relative to parenchyma evaluated across model variants. Single-feature exclusion variants systematically omit named marker genes or cell types, isolate single terms (Dens.Only, Expr.Only), apply original marker gene sets (MGS), or exclude all macrophage-related features (Mac-free) under identical cutoff thresholds without inflammatory filtering. Bars represent stage means and circles mark section-level data (b, c). n = 2 mice per stage. Fib, fibroblasts; IM, interstitial macrophages; SMC, smooth muscle cells; TM, *Trem2*^+^ macrophages; S0, S28, and S56, day 0, day 28, and day 56.

**Supplementary Fig. S4. Unsupervised spatial niche dissection identifies a pathognomonic lesion-associated cellular microenvironment.** **(a)** Spatial assignment of single cells to microenvironmental niches in representative sections across stages. **(b)** Cell-type composition across identified spatial niches, where dot diameter corresponds to the proportion of each cell lineage. **(c)** Relative abundance of microenvironmental niches across silica exposure stages. **(d)** Proportional distribution of cells within each niche stratified by intra-nodular versus parenchymal localization, pooled across the four exposed sections. **(e)** Lesion proportion of individual niches evaluated at day 28 and day 56. Bars display stage means and circles indicate section-level data (e). Scale bar, 500 µm (a). n = 2 mice per stage. AM, alveolar macrophages; AT1 and AT2, alveolar type 1 and type 2 cells; B, B cells; Cil, ciliated cells; Art-EC, Cap-EC, Lym-EC, and Ven-EC, arterial, capillary, lymphatic, and venous endothelial cells; CM, cardiomyocytes; DC, dendritic cells; EC, endothelial cells; EC-lowQC, low-quality endothelial cells; Fib, fibroblasts; IM, interstitial macrophages; Meso, mesothelial cells; NA, unassigned; PC, plasma cells; Peri, pericytes; Prolif, proliferating cells; SMC, smooth muscle cells; T, T cells; TM, *Trem2*^+^ macrophages; S0, S28, and S56, day 0, day 28, and day 56.

**Supplementary Fig. S5. Sensitivity analyses confirm robust intra-nodular *Trem2^+^* macrophage enrichment across segmentation parameters.** **(a–d)** Log_2_ fold enrichment of *Trem2*^+^ macrophages within lesions relative to parenchyma evaluated across systematic variations in posterior mixture cutoff **(a)**, kernel bandwidth **(b)**, spatial expression radius **(c)**, and nearest-neighbor search sizes **(d)**. Trend lines represent mean values across n = 2 biological replicates, with shaded regions reflecting replicate range; y-axis is truncated at 2.0. n = 2 mice per stage. TM, *Trem2*^+^ macrophages; S28 and S56, day 28 and day 56.

**Supplementary Fig. S6. Concentric radial mapping demonstrates internal *Trem2*^+^ macrophage positioning relative to mesenchymal populations.** **(a)** Mean normalized distance of six major cell types to lesion centroids in day 56 nodules, scaled from centroid (0) to 95th-percentile radius (1.0). **(b)** Paired comparison of mean normalized distance between *Trem2*^+^ macrophages and combined fibroblast/pericyte populations within individual sections. **(c)** Observed-to-expected spatial adjacency of fibroblasts and pericytes to *Trem2*^+^ macrophages stratified across concentric radial shells, with the outermost shell capturing cells beyond the 95th-percentile radius. Axis in (a) is truncated at 0.40; trend lines in (c) represent mean values across n = 2 biological replicates with shaded bounds, and dashed line marks random expectation. Shell ratios in (c) are compared with 500 label permutations within the same shell, and one-sided empirical P equals the number of permutations at or above the observed value plus one, divided by 501. n = 2 mice per stage. AM, alveolar macrophages; Fib, fibroblasts; IM, interstitial macrophages; Peri, pericytes; SMC, smooth muscle cells; TM, *Trem2*^+^ macrophages; S28 and S56, day 28 and day 56.

**Supplementary Fig. S7. Compartment-aware null models confirm random intra-nodular macrophage–fibroblast spatial proximity in exposed lungs.** **(a)** Observed-to-expected ratio of fibroblast and pericyte representation among *Trem2*^+^ macrophage neighbors evaluated across four distinct null permutation models. **(b, c)** Neighbor representation ratios in day 56 sections plotted across varying neighbor numbers (b) and spatial interaction radii (c) under global versus compartment-constrained nulls. Constrained null models permute cell labels strictly within nodular and parenchymal boundaries separately, within individual nodules, or within concentric radial distance bands surrounding centroids. Dashed lines denote theoretical random expectation. Bars represent mean values across n = 2 sections with circles marking section data (a); trend lines represent stage means with shaded bounds (b, c). Unexposed control sections are excluded due to low cell counts. Observed statistics are compared with 500 (a) or 200 (b, c) label permutations per null model, and one-sided empirical P equals the number of permutations at or above the observed value plus one, divided by the permutation count plus one. n = 2 mice per stage. TM, *Trem2*^+^ macrophages; S28 and S56, day 28 and day 56.

**Supplementary Fig. S8. Alveolar health index robustly discriminates silicosis stages across component weighting paradigms.** **(a)** Section-mean alveolar health index (AHI) values evaluated across exposure stages. **(b)** Stage-specific mean AHI scores calculated across six distinct component weighting schemes. **(c)** Spatial AHI profile plotted relative to distance from the nearest lesion centroid across individual exposed sections. **(d)** Spearman rank correlation between tile AHI values and tissue coverage across stages. Bars represent stage means and circles mark section-level data (a, d). n = 2 mice per stage. S0, S28, and S56, day 0, day 28, and day 56.

**Supplementary Methods**

**Animals and silicosis model**

Male C57BL/6 mice (8–10 weeks of age) were housed under specific-pathogen-free conditions and randomly assigned to three groups (n = 5 per group). Silicosis was induced under isoflurane anesthesia by a single intratracheal instillation of silica particles (20 nm mean diameter, purity ≥ 99%; Solarbio, Beijing, China) suspended in sterile saline (100 mg/mL, 50 µL per mouse), whereas unexposed control mice received an equivalent volume of sterile saline. At 0, 28, and 56 days post-instillation, animals were euthanized by intraperitoneal injection of pentobarbital sodium. The lungs were perfused with phosphate-buffered saline (PBS), inflated through a tracheal cannula with 10% neutral-buffered formalin (NBF), fixed in 10% NBF for 24 h at room temperature, embedded in paraffin blocks, and stored at room temperature until sectioning. A total of five animals per time point were modeled, and two representative biological replicates per stage were profiled by spatial transcriptomics, namely sections 337976 and 337996 for unexposed controls (day 0), 335012 and 338017 for early nodular fibrogenesis (day 28), and 342592 and 342598 for mature silicotic consolidation (day 56).

**Xenium sample preparation and tissue microarray construction**

All six lung specimens were assembled into a single 2 × 3 tissue microarray (TMA) for simultaneous *in situ* hybridization and imaging on a single slide. Formalin-fixed paraffin-embedded (FFPE) lung tissues from six representative blocks were arrayed into a recipient paraffin block by standard manual tissue microarray methods, with interstitial spaces backfilled with blank paraffin. The assembled block was briefly heated in a warm drawer (approximately 45 °C) to fuse the paraffin and even the block face, cooled to room temperature, and stored in a sealed desiccator at 4 °C. Workstations and equipment were cleaned with RNase AWAY (Thermo Fisher Scientific) followed by 70% isopropanol, and all solutions were prepared using nuclease-free reagents. Xenium profiling was performed by Hangzhou Cosmos Wisdom Biotechnology Co., Ltd. (Zhejiang, China). High-plex *in situ* profiling utilized the predesigned Xenium Mouse 5K Pan Tissue & Pathways Panel (10× Genomics) targeting 5,006 genes, supplemented with 40 negative-control probes, 21 genomic-control probes, and 8 deprecated codewords (5,075 targets in total with probe specifications). Key lineage markers and pathway components assayed in the panel included *Trem2*, *Gpnmb*, *Mmp12*, *Mmp14*, *Cd68*, *Col1a1*, *Col1a2*, *Fn1*, *Tgfb1*, *Marco*, *Mrc1*, *Nlrp3*, *Casp1*, *Pycard*, *Il18*, and *Cd44*. Canonical markers not included in the targeted library, including *Spp1*, *Lgals3*, and *Cd63* for macrophage activation and *Acta2*, *Il1b*, *Sftpc*, *Fabp4*, *Col3a1*, *Krt8*, and *Serpinb2*, were not measured, and markers present on the panel were used in their place.

**Xenium *in situ* hybridization and imaging**

FFPE sections (5 µm thickness) were mounted onto a single Xenium slide (10.45 mm × 22.45 mm, 10× Genomics), deparaffinized, and decross-linked according to 10× Genomics Demonstrated Protocols, with boundary staining against ATP1A1, CD45, and E-cadherin for cell segmentation. Probe hybridization was conducted for 18 h at 50 °C, followed by ligation for 2 h at 37 °C and rolling circle amplification for 2 h at 30 °C, with washes in PBS and PBS with Tween-20 (PBS-T). After amplification, nuclei were stained with DAPI and background autofluorescence was chemically quenched. Processed slides were stored in PBS-T in the dark at 4 °C prior to instrument loading. Regions of interest encompassing all six lung sections were selected from a low-resolution whole-slide overview, and optical fields of view across successive decoding cycles were aligned and stitched using the DAPI nuclear channel. During decoding, onboard analysis assigned a quality value to each decoded transcript.

**Postrun histology, image registration and regional overlay**

Following the Xenium run, slides were removed from the Analyzer and the quencher was removed according to 10× Genomics Demonstrated Protocol, and the profiled tissues were then subjected to post-Xenium hematoxylin and eosin (H&E) staining, including xylene (3 × 3 min), graded ethanol (100%, 95%, and 70%), hematoxylin staining, bluing, eosin counterstaining, and coverslipping. Subsequently, brightfield histological images were digitized on a slide scanner at 20× magnification. Landmark pairs were placed on the Xenium DAPI morphology image and the H&E image in Xenium Explorer (10× Genomics), an affine transform fitted to them by least squares registered the two images, and registered images were reviewed manually for visual artifacts. To correlate transcriptional architecture with tissue histology, regional histopathological overlays were generated by combining computational lesion segmentations with 2D kernel density estimates (kde2d, bandwidth h = 80 µm over a 150 × 150 grid spanning each section with a 50 µm margin). Within these overlays, nodule boundaries were established directly from the final MENS-defined lesions, designated as cellular nodules at day 28 and consolidated fibrotic nodules at day 56. The two labels distinguish stages and were not assigned from lesion morphology. Major anatomical structures were demarcated according to lineage-specific local densities. Specifically, bronchiolar regions were defined by ciliated-cell densities at or above the 90th percentile of per-cell density within the section. Vascular structures were identified by the densities of endothelial cells (all endothelial types except low-quality endothelial cells) and smooth muscle cells (SMC) at or above the 90th percentile. In parallel, alveolar parenchyma was mapped by alveolar type 1 (AT1) and type 2 (AT2) cell densities at or above the 70th percentile, accommodating the expansive spatial distribution of alveolar tissue. Finally, region labels were assigned in a hierarchical priority: nodule > bronchiole > vessel > alveoli > other.

**Quality filtering and data preprocessing**

Transcripts with a quality value below 20 were excluded from the cell counts by Xenium Onboard Analysis, and counts from control probes and codewords were held apart from the gene counts. Cells were delimited by the Xenium multimodal cell segmentation, 96.6% of retained cells by the interior stain, 2.4% by the boundary stain, and 1.1% by nuclear expansion. After segmentation, cells were assigned to the six sections by DBSCAN clustering of slide coordinates (eps = 100 µm, minPts = 100), which set aside tissue from other studies on the same slide and left 453,942 cells before filtering. Single cells were filtered by requiring ≥ 10 detected transcripts and removing cells exceeding the pooled 98th percentile of transcript counts across the six sections. The filter was applied in two passes, the first removing cells above 746 transcripts and retaining 433,026 cells and the second, computed on the retained cells, removing cells above 614 transcripts. Relative to the cells present before filtering, the upper bound removed 3.3% and 0.1% of cells in the day-0 sections, 3.8% and 1.3% at day 28, and 6.9% and 9.4% at day 56. Initial filtering yielded 50,985 and 47,624 cells at day 0, 92,298 and 115,902 cells at day 28, and 60,487 and 57,082 cells at day 56 (totaling 424,378 cells). High-resolution examination of post-run H&E images identified an extraneous, non-pulmonary tissue strip along the inferior margin of section 342598, separated from lung parenchyma by an acellular paraffin gap and composed of dense, small round cells (74.8% proliferating cells). The upper edge of the strip was traced column by column on the H&E image and fitted with a straight line (residual standard deviation 3.5 µm). Excluding the 705 cells beyond the edge or within 10 µm of it left 56,377 validated cells in section 342598 and 423,673 cells across the complete dataset. Sections retained their native slide coordinates, and multi-section figures offset each section at plotting time.

**Dimensionality reduction, clustering and cell-type annotation**

Transcript counts were log-normalized per cell (LogNormalize) with a scale factor equal to the median transcript count per cell (162) in Seurat v5, and 3,000 variable features were selected by the variance-stabilizing transformation (vst) method. Principal component analysis (PCA) was performed to compute 50 components, with the leading 30 components retained for the shared nearest-neighbor graph (k = 20) and the UMAP embedding, using the Seurat default seeds (42 for PCA and UMAP, 0 for clustering). Community detection was conducted using the Seurat default Louvain algorithm across resolutions from 0.1 to 0.8, which gave 11 to 29 clusters, and resolution 0.5, set in advance, was used (22 clusters). Each cluster was labeled as one of 22 cell types from its top markers, without lineage-level re-clustering, and lineage (epithelial, endothelial, immune, mesenchymal, or other) was assigned from cell type.

**MENS nodule identification**

To define fibrotic nodule boundaries directly from spatial transcriptomic measurements, we formulated the Multi-Evidence Nodule Score (MENS), coupling cellular co-localization with local fibrogenic transcript expression. The cell-density component was the 2D kernel density of *Trem2*⁺ macrophages, interstitial macrophages, fibroblasts, pericytes, and smooth muscle cells (kde2d, grid dimensions 200 × 200, bandwidth h = 100 µm, corresponding to a Gaussian standard deviation σ = 25 µm, over the extent of these cells padded by 50 µm), evaluated at the grid node of each cell. The transcriptional component was the summed log-normalized expression of *Cd68*, *Col1a1*, *Fn1*, and *Tgfb1* averaged over the focal cell and up to 29 of its nearest spatial neighbors lying within 50 µm (k-d tree search). Each component was min-max normalized to the unit interval [0, 1] within each section and summed with equal weighting. To accommodate inter-sample variations in cellularity without imposing arbitrary percentile quotas, a two-component Gaussian Mixture Model (GMM, scikit-learn, 10 initializations, seed 1234) was fitted to the combined MENS distribution. The candidate lesion threshold was defined as the infimum score above the lower component mean where the posterior probability of the upper component reached ≥ 0.5, incorporating a median guard requiring the cutoff to exceed the section median (satisfied across all sections). Candidate cells (MENS ≥ threshold) were partitioned into discrete spatial lesions using DBSCAN (scikit-learn, eps = 150 µm, minPts = 30), discarding noise cells and clusters of fewer than 30 cells. Lesion centroids were computed downstream as the mean coordinate of member cells. To prevent physiologically dense, non-inflamed bronchovascular sheaths from being misidentified as lesions in control sections, candidate clusters were evaluated against a binomial inflammatory infiltrate filter. A candidate cluster was confirmed as a bona fide lesion only if the combined proportion of *Trem2*⁺ macrophages, T cells, B cells, and plasma cells was significantly enriched over the section's non-candidate parenchymal baseline (one-sided binomial test, Benjamini–Hochberg false discovery rate q < 0.05). Resident alveolar macrophages, interstitial macrophages, and dendritic cells were excluded from the inflammatory criterion, given their physiological presence in normal peribronchovascular tissue. In unexposed control sections, no candidate cluster passed the test (smallest q = 0.29 and 0.23, with pooled inflammatory shares of 3.4% and 4.6% against 4.2% and 5.7% among non-candidate cells), yielding zero confirmed lesions. In exposed lungs, the inflammatory filter excluded 6.0% of the cells in candidate clusters at day 28 and 0.13% at day 56. The Python implementation used for lesion labeling reproduced the MENS values of the original R implementation at Pearson r = 0.991 to 0.998 across sections.

**Spatial niche analysis**

To dissect microenvironmental architecture across pulmonary tissue, spatial niches were resolved using the BuildNicheAssay framework in Seurat v5. For each cell, local microenvironmental context was characterized by cell-type counts among its 20 nearest spatial neighbors, the cell itself included (neighbors.k = 20). Unsupervised k-means clustering of the scaled neighborhood matrix (niches.k = 10, 30 random starts, no dedicated seed), fitted once to all 424,378 cells before removal of the non-pulmonary strip, grouped spatial neighborhoods into ten cellular niches. The number of niches was fixed in advance, and no other number was tested. Cell-type compositions and niche frequencies were quantified across sections, and the share of each niche's cells located inside lesions was computed for each exposed section and pooled across the four exposed sections.

**Macrophage subtype analysis**

Macrophage subpopulations corresponded to three of the 22 clusters, labeled from their top markers as resident alveolar macrophages (*Chil3*, *Atp6v0d2*, and *Lpl*), interstitial macrophages (*Csf1r*, *Adgre1*, and *F13a1*), and *Trem2*⁺ macrophages (*Gpnmb*, *Il1rn*, and *Itgb2*). The labels were checked against *Marco*, *Mrc1*, and *Pparg*, against *Ccr2* and *Cx3cr1*, and against *Trem2*, *Gpnmb*, and *Mmp12*, respectively. For display, counts were pooled within each subtype and scaled to 10,000 transcripts, and genes detected in at least 10% of the subtype's cells and above 0.5 counts per 10,000 were ranked by the share of macrophage signal carried by the subtype. The checking markers of each subtype were then filled to four genes from the ranking, adding *Chil3* for alveolar macrophages, *F13a1* and *Aif1* for interstitial macrophages, and *Acod1* for *Trem2*⁺ macrophages. *Spp1*, *Lgals3*, *Cd63*, and *Ctsd* are absent from the panel and were not used. Spatial localization relative to nodular architecture was determined in the exposed sections by calculating the Euclidean distance from each macrophage coordinate to the nearest lesion centroid, the mean coordinate of each final lesion.

**Mesenchymal pseudotime analysis**

To characterize mesenchymal cells during fibrogenesis, the mesenchymal subset (fibroblasts, pericytes, smooth muscle cells, and mesothelial cells, 82,676 cells in total) was compared by the share of each subtype in the mesenchyme of each section. In the exposed sections, the share of each subtype located inside lesions was compared with the share of all cells inside lesions of the same section. For trajectory inference, the subset was scaled and re-analyzed using PCA (20 components computed) and UMAP embedding over dimensions 1 to 15. The processed Seurat object was converted into a SingleCellExperiment object, and differentiation trajectories were inferred using Slingshot in the 20-component PCA space with cell types as clusters, specifying quiescent fibroblasts as the root state. Slingshot returned three lineages, ending in mesothelial cells, pericytes, and smooth muscle cells, and each cell took its minimum pseudotime across lineages. Local polynomial regression (LOESS) of UMAP coordinates against pseudotime served only to draw the lineage curves. Fibroblast pseudotime decreased with exposure and was lower inside lesions than in parenchyma, and a similar offset appeared in the bronchovascular candidate regions of the control sections, which carry no lesion. The pseudotime ordering was therefore not used further and is summarized per section and cell type without a test between stages.

**Grid-based alveolar analysis and Alveolar Health Index**

To assess continuous parenchymal injury across lung tissue, sections were partitioned into regular square spatial tiles measuring 200 µm × 200 µm, excluding sparsely populated tiles containing fewer than five cells. An exploratory Alveolar Health Index (AHI) was formulated to evaluate the microenvironmental integrity of alveolar parenchyma. The index integrated the AT1 fraction (f_AT1), the AT2-to-AT1 balance r = min(1, max(0, log₂[(AT2 + 1)/(AT1 + 1)] / 3)), and the immune fraction (f_immune, all immune-lineage cells), with fractions taken over all cells in the tile. The index was computed as AHI = w₁ · f_AT1 + w₂ · (1 − r) + w₃ · (1 − f_immune) and bounded within the unit interval [0, 1]. To verify that findings were robust to component weighting, six distinct weighting schemes (w₁, w₂, w₃) were systematically compared, namely the baseline formulation (0.4/0.3/0.3), equal weights (1/3, 1/3, 1/3), AT1-predominant (0.6/0.2/0.2), immune-predominant (0.2/0.2/0.6), balance-predominant (0.2/0.6/0.2), and AT1 proportion alone, and tile AHI was compared with tile tissue coverage by Spearman correlation. Spatial injury gradients were mapped across 100-µm bands of distance from the tile center to the nearest lesion centroid, below 900 µm in the main-figure profiles and up to a final band pooling all tiles at 1,100 µm or more in the supplementary profile. For each exposed section, the Pearson correlation between band-mean AHI and band distance was computed below 900 µm.

**Pathway composite scoring**

To overcome transcript sparsity and provide robust spatial measurements of cellular signaling, composite pathway scores were calculated across all 423,673 analyzed cells. Log-normalized expression of each gene was standard-normalized (z-scored) across the pooled cells prior to calculating the per-cell arithmetic mean across signature genes, ensuring cross-section comparability. Signatures comprised TGF-β signaling (*Tgfb1*, *Tgfbr1*, *Tgfbr2*, *Smad2*, *Smad3*), NLRP3 inflammasome activation (*Nlrp3*, *Casp1*, Gsdmd, *Il18*, *Pycard*), and extracellular matrix remodeling (*Mmp2*, *Mmp7*, *Mmp9*, *Mmp12*, Timp1, Lox). For spatial visualization, color ramps ran from 0 to the pooled 99.3rd percentile of each score, with cells displaying non-positive scores rendered in neutral light gray to highlight enrichment patterns.

**Spatial proximity analysis**

Spatial proximity between *Trem2*⁺ macrophages and mesenchymal cells (fibroblasts and pericytes) was evaluated per section as the fraction of fibroblasts and pericytes among the 10 nearest neighbors of each *Trem2*⁺ macrophage, the macrophage itself excluded. Distance-based neighborhoods (25–100 µm) were examined only in the parameter sensitivity analysis. To obtain an observed-to-expected ratio, the observed mean fraction was divided by its mean under label permutation, and under the global permutation the expected fraction equals the section-wide share of fibroblasts and pericytes. For each section, cellular phenotypes were permuted randomly 500 times (random seed 1234). Empirical one-sided P values were determined as P = (r + 1) / (N + 1), where r is the number of permutations with a mean fraction greater than or equal to the observed value and N = 500. Unexposed control sections (337976 and 337996) were excluded from proximity testing due to the scarcity of *Trem2*⁺ macrophages (17 and 32 cells, below the minimum of 50 per section), which precludes robust spatial interaction modeling.

**Leave-one-feature-out nodule definition**

To verify that MENS-defined nodular boundaries and subsequent *Trem2*⁺ macrophage enrichment were not artifacts of circular feature selection, a leave-one-feature-out (LOFO) sensitivity ablation was conducted across 14 feature configurations. These models included the primary full model, the five density cell types combined with the expression genes named in the originally submitted Methods (*Col1a1*, *Col1a2*, *Fn1*, *Trem2*, and *Gpnmb*), isolated density-only and expression-only models, five single-cell-type dropout models (each iteratively omitting one density lineage), and four single-gene dropout models (each iteratively omitting *Cd68*, *Col1a1*, *Fn1*, or *Tgfb1*). Furthermore, an entirely macrophage-free formulation was constructed, restricted exclusively to mesenchymal cell density (quiescent fibroblasts, pericytes, and smooth muscle cells) and non-macrophage matrix transcripts (*Col1a1*, *Fn1*, and *Tgfb1*). Each variant score was segmented by refitting the section-specific two-component GMM with the cutoff rule of the primary pipeline, and variants were evaluated on candidate cells, before DBSCAN and the inflammatory filter (which specifically references *Trem2*⁺ macrophages). Robustness was evaluated via two complementary criteria: boundary concordance (Jaccard similarity index comparing the candidate cells of each variant with those of the primary model) and biological enrichment (the intra-nodular cellular percentage and log₂ fold enrichment of *Trem2*⁺ macrophages relative to all cells below the cutoff).

**Spatially constrained permutation**

To evaluate the spatial organization of *Trem2*⁺ macrophages relative to mesenchymal cells beyond random expectation, three compartment-aware spatial null models were constructed alongside global permutations (500 iterations per section, random seed 1234, evaluated over the 10 nearest neighbors of each *Trem2*⁺ macrophage). In the global unconstrained permutation model, phenotype labels were permuted randomly across all cells within the section to test for tissue-wide spatial association. In the compartment-constrained strata model, cell labels were shuffled strictly within matching anatomical zones (permuting intra-nodular cells exclusively among intra-nodular cells, and parenchymal cells strictly within parenchyma), preserving macroscopic lesion compartmentalization. In the intra-nodular permutation model, analysis was restricted to lesion cells and nearest neighbors were recomputed among them. Cell labels were then permuted strictly within individual lesions (permuting within each unique lesion identifier separately, with at least 50 *Trem2*⁺ macrophages inside lesions per section), preserving internal lesion cell counts and local geometries. Finally, in concentric radial distance strata, cell labels were shuffled strictly within 100-µm concentric distance shells (from 0–100 µm to 1,900–2,000 µm of distance to the nearest lesion centroid), controlling for radial cell density gradients. Empirical P values were calculated as P = (r + 1) / (N + 1), where r is the number of permutations in which the null neighbor fraction equaled or exceeded the observed fraction, and N = 500. The lesion and radial strata were defined by the final lesion labels after the inflammatory filter.

**Radial position within nodules**

To determine the internal microarchitectural organization of cellular lineages across mature lesions, spatial coordinates of intra-nodular cells were mapped relative to nodule geometry. Lesions containing fewer than 30 cells were excluded from radial profiling. Geometric centroids were calculated as the bivariate mean coordinate of all constituent lesion cells. To accommodate variations in nodular dimensions, the Euclidean distance of each cell to its corresponding centroid was normalized to the 95th-percentile radius of the lesion (clamped at 1.5 to accommodate peripheral boundary cells). Normalized radial distances were partitioned into five concentric shells, spanning core (0–0.25), inner ring (0.25–0.50), intermediate ring (0.50–0.75), outer boundary (0.75–1.00), and outer tail (> 1.00, lesion cells beyond the 95th-percentile radius). The per-animal contrast between fibroblasts with pericytes and *Trem2*⁺ macrophages required at least 50 cells of each inside the lesions of a section. Shell-specific spatial adjacency of fibroblasts and pericytes to *Trem2*⁺ macrophages was tested over the 10 nearest lesion-cell neighbors by permuting cell labels among lesion cells strictly within each concentric shell (exposed sections only, requiring ≥ 30 *Trem2*⁺ macrophages per shell and ≥ 50 per section, 500 permutations, random seed 1234).

**Morphology-only validation of the nodule boundary**

To provide an independent, transcript-free histological benchmark for computational lesion definition, morphological parameters derived from the Xenium Onboard multimodal cell segmentation were evaluated without molecular or cell-type inputs. Morphometric features (segmented cell area and nucleus area) were extracted from the segmentation output (cells.parquet), and all 423,673 analyzed cells matched a segmentation record. Tissue sections were partitioned into 100 µm × 100 µm grid tiles (retaining tiles with ≥ 5 cells). Three morphology-only parameters were quantified per tile, comprising nuclear density (segmented cells per mm², 99.6% of which hold one nucleus), mean nuclear area (µm²), and tissue area coverage (cumulative segmented cell area divided by total tile area, bounded at 1.0). Ground-truth lesion classification was assigned to tiles containing ≥ 50% MENS-confirmed lesion cells. Receiver Operating Characteristic (ROC) curves and Areas Under the Curve (AUC) were computed to evaluate the capacity of transcript-free morphological metrics to distinguish fibrotic lesions from surrounding parenchymal lung tissue. Overlap with a nuclear-density reference was quantified as the Dice coefficient between lesion tiles and an equal number of tiles with the highest nuclear density.

**Parameter sensitivity**

To assess the robustness of nodular boundary definition and cellular enrichment, systematic one-at-a-time parameter sensitivity analyses were performed across four operational dimensions. These dimensions comprised GMM posterior probability cutoffs (0.3, 0.4, 0.5, 0.6, and 0.7), kernel density bandwidths (h = 50, 75, 100, 150, and 200 µm), local gene-expression radii (25, 50, and 75 µm), and nearest-neighbor pool sizes (k = 15, 30, and 50). At every parameter combination, the binomial inflammatory infiltrate filter was applied. Spatial proximity of fibroblasts and pericytes to *Trem2*⁺ macrophages was similarly re-evaluated across varying neighborhood sizes (k = 5, 10, 20, and 30) and spatial interaction radii (25, 50, 75, and 100 µm, searching the 50 nearest cells) under both global and compartment-constrained null models (200 permutations per setting, random seed 1234). Unexposed control sections, which held 17 and 32 *Trem2*⁺ macrophages, were excluded from sensitivity comparisons.

**Statistical analysis**

Computational and statistical analyses were performed using R (version 4.5.2) utilizing packages Seurat (v5.4.0), MASS, RANN, dbscan, slingshot, SingleCellExperiment, and ggplot2, and Python (version 3.10.12) utilizing numpy, pandas, scipy, and scikit-learn. A fixed random seed (1234) was used for the GMM expectation-maximization initializations and the spatial null permutations, PCA, UMAP, and Louvain clustering used the Seurat default seeds, and the niche k-means used no dedicated seed. Multiple hypothesis testing corrections were applied using the Benjamini–Hochberg false discovery rate procedure where indicated, with significance defined at *q* < 0.05. Empirical permutation *P* values were calculated as *P* = (*r* + 1) / (*N* + 1), where *r* is the count of null permutations exceeding or matching the observed statistic and N is the total permutation count. To prevent pseudo-replication, statistical inferences were based strictly on biological replicates, with stage-level summary metrics reported as individual animal measurements (*n* = 2 per stage) or their respective ranges. Correlations were computed as Spearman rank coefficients except where a Pearson coefficient is stated.
